# Ranking the Impact of Different Tests on a Hypothesis in a Bayesian Network

**DOI:** 10.3390/e20110856

**Published:** 2018-11-07

**Authors:** Leila Schneps, Richard Overill, David Lagnado

**Affiliations:** 1 Institut de Mathématiques de Jussieu, Paris 75013, France; 2 Department of Informatics, King’s College London, London WC2R 2LS, UK; 3 Department of Experimental Psychology, University College London, London WC1H 0AP, UK

**Keywords:** Bayesian networks, impact measures, Kullback–Leibler divergence, tornado method

## Abstract

Testing of evidence in criminal cases can be limited by temporal or financial constraints or by the fact that certain tests may be mutually exclusive, so choosing the tests that will have maximal impact on the final result is essential. In this paper, we assume that a main hypothesis, evidence for it and possible tests for existence of this evidence are represented in the form of a Bayesian network, and use three different methods to measure the impact of a test on the main hypothesis. We illustrate the methods by applying them to an actual digital crime case provided by the Hong Kong police. We conclude that the Kullback–Leibler divergence is the optimal method for selecting the tests with the highest impact.

## 1. Introduction

In this article, we consider the situation of a hypothesis whose probability can be updated given the results of a series of tests. Our work is motivated by the fact that this situation arises very typically in the course of criminal investigations, when the initial hypothesis concerns the guilt of a suspect, and actual forensic testing is to be applied to material pieces of evidence. Our goal is to rank the possible tests in order of the impact they will have on the probability of guilt. Such a ranking has useful applications, as we will show on a concrete example, in cases where one can perform a great number of different tests at significant cost of time and money, and one would like to begin with the most important ones, and exclude those that can only have minimal impact at best. Similarly, the method can be useful to decide which test to perform in cases where one must choose between one of two possible tests, for example between determination of the mixed DNA profile or of the Y-haplotype in the case of a low-template mixed DNA sample from a murder victim, on which only one reliable test can be performed due to the limited quantity.

In order to give a rigorous definition of the notion of impact, we assume that the evidence is represented in the form of a Bayesian network (cf. [1,2] for a basic reference) with one particular node selected as the main hypothesis, and a collection of terminal nodes (nodes with no children) corresponding to specific forensic tests, the result of each of which has the power to modify and update the probability of the main hypothesis. We will establish a measure of impact of each test in such a way that, at each stage of testing, one can determine which is the best next test to perform for maximal impact. This approach also naturally yields the notion of a *threshold of usefulness* below which the remaining tests are not sufficiently useful to justify performing them, which will be applicable in situations where there are many possible tests but not enough time, resources or material to perform them all.

The general notion that acquiring new information updates the probability of a hypothesis is not new. It was the original motivation for Bayes’ theorem (proved by Bayes in 1763), and underlies the recent push to use the theorem in the context of criminal inquiries in order to quantify just how much effect a given fact actually has on the hypothesis of guilt; to give, in other words, a mathematical meaning to the phrase “weight of evidence”. Similarly, the notion of entropy (which we recall below) is used to measure the result of new information or knowledge in the form of reduction in doubt, or reduction in uncertainty [3]. These questions are related to the psychology of investigation and decision-making and the study of optimal strategies, which are the focus of intensive study (see, for example, [4,5]).

The novelty of our approach in this paper consists in bolstering the investigator’s intuitive approach by assessing the importance of a piece of evidence *before* any tests on it have been performed, thus before any actual new fact is added to the investigator’s knowledge. Our measure of impact is based only on the probabilities of the various possible outcomes of testing, and provides a concrete algorithm for the investigator to select the tests to be performed with highest priority.

Because our first step must be to define the notion of the impact of an unperformed test, we began by investigating various different methods. In Section 2 below, we introduce three possible methods for assessing the impact of a test on the selected hypothesis:

- the *expected information gain method*, based on the Kullback–Leibler formula for information gain [6],
- the *tornado* method [7],
- the *single missing item* method [8].

In Section 3, we discuss the advantages and disadvantages of each method on theoretical examples, and argue that the expected information gain method has a distinct advantage over the other methods. In Section 4, we apply and compare the methods on a specific example of a type of crime studied by the Hong Kong Police Department: illegal file sharing using Bit Torrent. In this type of crime, a user performs a series of digital operations, each of which leaves a possible trace on his computer (according to the ability of the user to erase his digital tracks) that may be detected by digital forensic analysis. From an observation of the procedure used by Hong Kong police to examine a computer seized on suspicion of this type of crime [9,10] together with interviews with thirty-one experienced digital forensic examiners, the authors of [11] established a Bayesian network model for the digital investigation, showing that there are 18 forensic tests that can be performed to verify five subhypotheses, each of which supports the main hypothesis that the suspect’s computer was used for file sharing using Bit Torrent. Our analysis in Section 4 is based on their Bayesian network.

## 2. Three Methods to Choose the Most Important Test

Consider a Bayesian network with discrete-valued nodes, equipped with the following extra data:

- One distinguished node that we call *G*; item A subset of terminal nodes, which we call $E_{1},\ldots ,E_{m}$;
- A given state for all the other nodes of the network, which can be either fixed to one of their values, or unfixed.

We may think about the selected node *G* as the “guilt node” and the chosen terminal nodes as “test nodes” corresponding to various actual forensic tests that can be performed, and whose results will modify the probabilities of the hypotheses of the node *G* (“guilt”).

Given this data, we will discuss three different methods to assess the impact that each test will have on the selected node. These methods produce an ordering of tests by impact as follows: one starts by measuring the impact of each test, then one performs the test with maximal impact, inserts the result into the Bayesian network, and starts the process again, using the method to assess the impact of each test, selecting the most useful one, performing it, etc. We note that, except for the third method presented below, the most useful test at any stage may depend on the actual result of the previous test. Although one may use our methods to produce a ranking of tests before beginning the testing, the ranking of later tests may not be optimal given the results of the earlier tests, so we do not recommend using the methods in this manner (except for the third one).

The first method is the one necessitating the longest explanation, as we give a short introduction to the Kullback–Leibler formula.

### 2.1. The Expected Information Gain Method

For 1≤i≤m, let $e_{i}$ be the number of values of the node $E_{i}$, and let *g* denote the number of values of *G* (for instance, if the values of *G* are true/false, then g=2). We write G(1),…,G(g) for the values of the node *G*, and $E_{i}\left(1\right),\ldots ,E_{i}\left(e_{i}\right)$ for the values of the node $E_{i}$.

We first give the Kullback–Leibler formula, which computes the information gain on the node *G* associated to each possible result of each test, i.e., to each given outcome $E_{i}\left(j\right)$, 1≤i≤m, $1\leq j\leq e_{i}$. We write $I_{i,j}^{G}$ for this information gain; it is the *Kullback–Leibler divergence from the distribution $P\left(G(k)\right)$ to $P\left(G\left(k\right)|E_{i}\left(j\right)\right)$*, given by

$$I_{i,j}^{G}=\sum_{k=1}^{g}P\left(G\left(k\right)|E_{i}\left(j\right)\right)\;\log_{g}\left(\frac{P\left(G\left(k\right)|E_{i}\left(j\right)\right)}{P(G(k))}\right).$$

Note that we choose to use the base *g* logarithm, which scales the result so that the information gain in the case where the initial state is zero knowledge (maximal entropy) and the final state is absolute knowledge (zero entropy), is equal to 1. We note that, if the initial state is not one of zero knowledge, then the information gain can be greater than 1, which indicates that a prior estimate has been reversed. For instance, if g=2 and

$$P\left(G(1)\right)=0.1,\;\;P\left(G(2)\right)=0.9$$

are the prior probabilities, then, if after performing the test $E_{i}$ and obtaining the result $E_{i}\left(j\right),$ we have posterior probabilities

$$P\left(G\left(1\right)|E_{i}\left(j\right)\right)=0.2,\;\;P\left(G\left(2\right)|E_{i}\left(j\right)\right)=0.8,$$

the information gain will be 0.064, but, if instead the posterior probabilities are given by

$$P\left(G\left(1\right)|E_{i}\left(j\right)\right)=0.8,\;\;P\left(G\left(2\right)|E_{i}\left(j\right)\right)=0.2,$$

strongly reversing the priors, we find that the information gain is 2.536.

**Lemma** **1.** *The information gain $I_{i,j}^{G}$ is always positive.*

**Proof.**  This follows from applying Jensen’s formula to the log function; for a finite collection $a_{k}$ of positive real numbers and a finite collection $h_{k}$ of real numbers, we have Jensen’s inequality

$$\frac{\sum_{k}a_{k}\;\log_{g}\left(h_{k}\right)}{\sum_{k}a_{k}}\leq \log_{g}\left(\frac{\sum_{k}a_{k}h_{k}}{\sum_{k}a_{k}}\right).$$

In our case, for each fixed pair (i,j) with 1≤i≤g and $1\leq j\leq e_{i}$, we take

$$a_{k}=P\left(G\left(k\right)|E_{i}\left(j\right)\right),\;\;\;k=1,\ldots ,g$$

and

$$h_{k}=\frac{P(G(k))}{P\left(G\left(k\right)|E_{i}\left(j\right)\right)},\;\;\;k=1,\ldots ,g.$$

With these values, we have $\sum_{k=1}^{g}a_{k}=1$, so Jensen’s formula simplifies to

(1) $$\sum_{k=1}^{g}a_{k}\;\log_{g}\left(h_{k}\right)\leq \log_{g}\left(\sum_{k=1}^{g}a_{k}h_{k}\right).\;$$

The left-hand side of (1) is equal to $-I_{i,j}^{G}$, and the right-hand side of (1) simplifies to

$$\log_{g}\left(\sum_{k=1}^{g}P\left(G(k)\right)\right)=\log_{g}\left(1\right)=0.$$

Thus, $-I_{i,j}^{G}\leq 0$, which proves the claim. □

We can now define the *expected information gain $I_{E_{i}}^{G}$ on G from $E_{i}$* to be the sum of the information gain terms $I_{i,j}^{G}$, weighted by the probabilities of the results $E_{i}\left(j\right)$, i.e., we set

(2) $$I_{E_{i}}^{G}=\sum_{j=1}^{e_{i}}P\left(E_{i}\left(j\right)\right)I_{i,j}^{G}=\sum_{j=1}^{e_{i}}\sum_{k=1}^{g}P\left(E_{i}\left(j\right)\right)P\left(G\left(k\right)|E_{i}\left(j\right)\right)\;\log_{g}\left(\frac{P\left(G\left(k\right)|E_{i}\left(j\right)\right)}{P(G(k))}\right).\;$$

The expected information gain $I_{E_{i}}^{G}$ is in fact equal to the difference between the expected entropy of *G* after applying the test $E_{i}$, given by

$$\sum_{j=1}^{e_{i}}\sum_{k=1}^{g}P\left(E_{i}\left(j\right)\right)P\left(G\left(k\right)|E_{i}\left(j\right)\right)\;\log_{g}\left(P(G\left(k\right)|E_{i}\left(j\right))\right)$$

and the prior entropy of *G* before performing the test, given by

$$\sum_{k=1}^{g}P\left(G(k)\right)\;\log_{g}\left(P(G(k))\right).$$

The Kullback–Leibler formula measures the divergence between two probability distributions on the *G* hypothesis: the prior $P\left(G(k)\right)$ (probability of guilt before testing) and $P\left(G\left(k\right)|E_{i}\left(j\right)\right)$ (probability of guilt after performing the test $E_{i}$ and obtaining the result $E_{i}\left(j\right)$).

### 2.2. The Tornado Method

When the nodes *G* and $E_{i}$ are Boolean (or even if they are not Boolean, but have True and False among their possible values), a cruder method called the tornado method (This method is implemented, for example, in the AgenaRisk Bayesian network software [12], pp. 99–102.) can be used to rank the tests $E_{i}$. The tornado method consists of computing the impact of a test $E_{i}$ on the truth or falsehood of *G* as the difference

(3) $$T_{E_{i}}^{G}=P\left(G=\mathrm{True}|E_{i}=\mathrm{True}\right)-P\left(G=\mathrm{True}|E_{i}=\mathrm{False}\right)\;$$

and taking the most important test to be the one for which this impact is maximal (The name tornado arises because graphing the impacts as horizontal bars along the real axis from largest to smallest creates a tornado shape).

### 2.3. The Single Missing Item Method

Again, this method is best adapted to the Boolean situation but can be used if True and False are among the node values. This method relies on assessing how much information is lost if all the tests are positive except for one, as opposed to all the tests being positive. The formula is given by

(4) $$S_{E_{i}}^{G}=P\left(G=\mathrm{True}|E_{i}=\mathrm{True}\;\mathrm{for}\;\mathrm{all}\;i\right)-P\left(G=\mathrm{True}|E_{i}=\mathrm{False},\;E_{j}=\mathrm{True}\;\mathrm{for}\;j\neq i\right).\;$$

## 3. Comparison of the Methods

In this section, we will give an analytical comparison of the three methods, and argue using theoretical cases that the expected information gain method using the Kullback-Leibler formula is the best one. One of the reasons for this is simply that the other two methods share one important feature which diminishes their validity significantly in situations where the Bayesian network under consideration is not Boolean: namely, they take into account only the positive or negative values of each test. The more a panoply of tests may have non-Boolean outcomes, and, in particular, the larger the probabilities of the outcomes that are not strictly positive or negative, the more information from the network is ignored by these two methods.

### 3.1. Comparison of the Expected Information Gain Method with the Tornado Method

The expected information gain can represent a significant improvement over the tornado method in certain cases. To see this, consider a toy situation in which one could apply one of two possible tests $E_{1}$ and $E_{2}$ to a hypothesis *H*. All three nodes take only True/False values, and we assume that the prior probabilities on *H* are

P(H=True)=P(H=False)=0.5.

One situation where the expected information gain is significantly better than the tornado method is when $E_{1}$ is a test that rarely takes the value True but is very probative when it takes the value True. For instance, let the conditional probability table of $E_{1}$ be

$$\left\{\begin{array}{c} P(E_{1}=\mathrm{True}|H=\mathrm{True})=0.1,\\ P(E_{1}=\mathrm{False}|H=\mathrm{True})=0.9,\\ P(E_{1}=\mathrm{True}|H=\mathrm{False})=0.01,\\ P(E_{1}=\mathrm{False}|H=\mathrm{False})=0.99. \end{array}\right.$$

This gives $P(E_{1}=\mathrm{True})=0.055$, $P(E_{1}=\mathrm{False})=0.945$.

For $E_{2}$, instead, we will assume a more balanced probability of obtaining the value True or False, taking the conditional probability table CPT

$$\left\{\begin{array}{c} P(E_{2}=\mathrm{True}|H=\mathrm{True})=0.7,\\ P(E_{2}=\mathrm{False}|H=\mathrm{True})=0.3,\\ P(E_{2}=\mathrm{True}|H=\mathrm{False})=0.5,\\ P(E_{2}=\mathrm{False}|H=\mathrm{False})=0.5, \end{array}\right.$$

which gives the probabilities $P(E_{2}=\mathrm{True})=0.6$, $P(E_{2}=\mathrm{False})=0.4$.

Using the tornado method, we have

$$P\left(H=\mathrm{True}|E_{1}=\mathrm{True}\right)-P\left(H=\mathrm{True}|E_{1}=\mathrm{False}\right)=0.90909-0.47619=0.4329,$$

$$P\left(H=\mathrm{True}|E_{2}=\mathrm{True}\right)-P\left(H=\mathrm{True}|E_{2}=\mathrm{False}\right)=0.58333-0.375=0.20833,$$

so the impact of $E_{1}$ is significantly higher than that of $E_{2}$. However, it is immediately obvious in a practical sense that, if only one test can be done, it is much more likely to be useful to perform $E_{2}$ since $E_{1}$ has over 90% chance of coming up False, which gives almost no information with respect to the prior P(H)=0.5, since $P(H=\mathrm{True}|E_{1}=\mathrm{False})=0.4762$.

The expected information gain method yields a result much closer to this intuitive reasoning; namely, we have

$$I_{E_{1}}^{H}=0.002147,\;\;\;\;\;I_{E_{2}}^{H}=0.021,$$

so that the expected effect of the second test is, by this measure, ten times greater than the first one.

### 3.2. Comparison of the Expected Information Gain Method with the Single Missing Item Method

These two methods yield quite different results, which is not surprising as they are not measuring precisely the same thing. The single missing item method determines the difference to the probability of guilt of the absence of each item of evidence (a negative test result) if all the other items of evidence are present (positive test results). Its impact on the probability of guilt can be viewed as a crude measure of its probative value.

The weakness of this method for measuring the impact of a given item of evidence is that it does not take into account the probability, which can be significant, of the absence of other items of evidence (negative test results). This can skew the impact results similarly to the Tornado method. For example, in the previous example, the impact of $E_{1}$ is equal to 0.04 and the impact of $E_{2}$ is just 0.07424. The greater the likelihood that the tests will produce non-positive results, the greater the possibility that the single missing item method, which relies only on positive test results for the remaining items, may give rise to unrealistic impact results.

### 3.3. Comparison of the Tornado and Single Missing Item Methods

Since we have already shown that the expected information gain is superior to the other two methods, we do not need to make a direct comparison between them. However, it may be useful to briefly describe their difference. Since both methods only take Boolean values into account, we may assume that we have a Boolean network. The impacts on the guilt hypothesis of these two methods are actually measuring very different things, since the single missing item method makes the simple (static) assumption that the outcomes of all other evidential tests are positive, whereas the tornado method is dynamic in its treatment of the possible outcomes of the remaining evidential tests. Depending on the situation, it is not possible to say that one is better than the other. To see the difference, consider the simple network of the previous form, with a main node *H* connected to two test nodes $E_{1}$ and $E_{2}$, which are independent from each other.

Imagine a situation with 50–50 priors on *H*, such that $P(H=T|E_{1}=T)=0.7$ and $P(H=T|E_{1}=F)=0.3$, whereas $P(H=T|E_{2}=T)=0.8$ and $P(H=T|E_{2}=F)=0.4$, so that $E_{1}$ and $E_{2}$ have identical impact according to the tornado method. One can solve Bayes formula using these probabilities and find that they correspond to the following conditional probability table (CPTs):

$$\left\{\begin{array}{c} P(E_{1}=\mathrm{True}|H=\mathrm{True})=0.7,\\ P(E_{1}=\mathrm{False}|H=\mathrm{True})=0.3,\\ P(E_{1}=\mathrm{True}|H=\mathrm{False})=0.3,\\ P(E_{1}=\mathrm{False}|H=\mathrm{False})=0.7, \end{array}\right.$$

and

$$\left\{\begin{array}{c} P(E_{2}=\mathrm{True}|H=\mathrm{True})=0.4,\\ P(E_{2}=\mathrm{False}|H=\mathrm{True})=0.6,\\ P(E_{2}=\mathrm{True}|H=\mathrm{False})=0.9,\\ P(E_{2}=\mathrm{False}|H=\mathrm{False})=0.1. \end{array}\right.$$

If we then use these values to compare the two tests using the single missing item method, we find that $E_{2}$ has more impact than $E_{1}$. In this instance, this result may correspond more to intuition in the sense that, if one had to choose between $E_{1}$ and $E_{2}$, one might choose to go for $E_{2}$ in the hopes of getting the maximal possible probability result of 80% chance that H=T (i.e., that the suspect is guilty) if $E_{2}=T$, a level of certainty that none of the other possibilities affords. However, intuition is difficult to quantify and there can be other examples where the opposite can hold.

## 4. The Bit Torrent Case

### 4.1. Description of the Case

When illegally sharing a pirated copy of a film using the Bit Torrent peer-to-peer protocol, a user must go through several steps involving copying the film from an optical disk (DVD) to his computer, storing it in a digitized format, creating a .torrent file from the film file, publishing it online by messaging on newsgroups or other media, and activating the .torrent file (seeding). Furthermore, if another user downloads the uploaded film, this action will also leave digital traces on the source computer.

**Figure 1:**
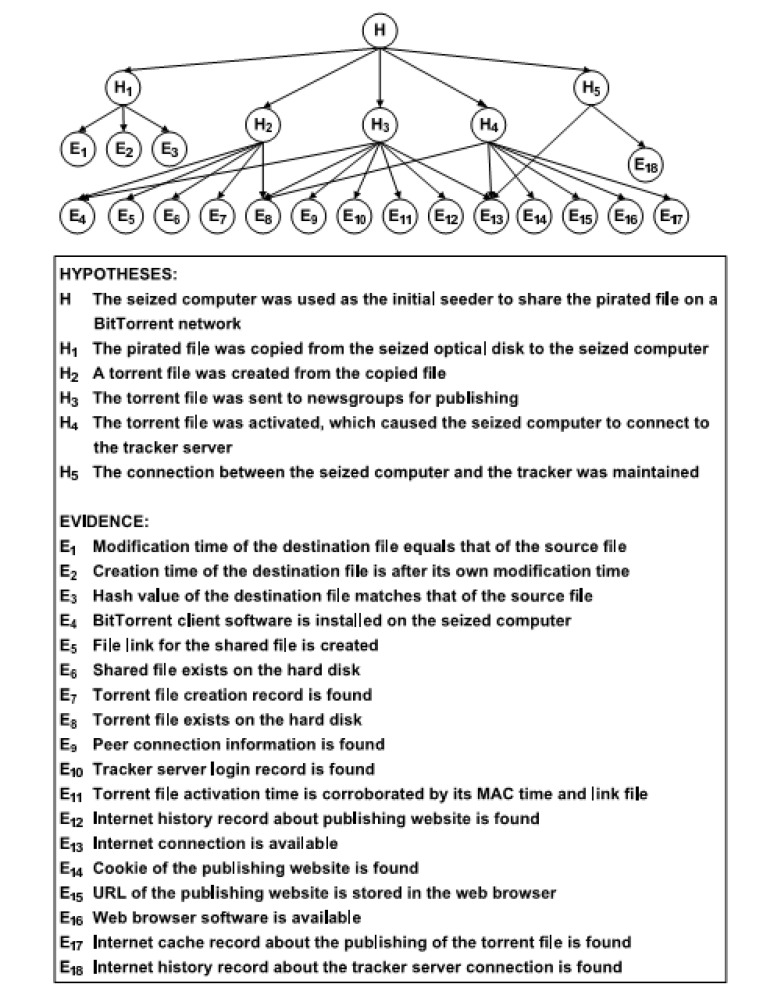
The Bit Torrent Bayesian network.

A set of 18 digital forensic tests to search a suspect’s computer for evidence of these activities was developed by the Hong Kong police. According to the description given in [10,11], starting from the hypothesis *H* that the seized computer was used for this type of illegal file sharing, the forensic analyst will attempt to validate five hypotheses, by performing at least some of the 18 tests to check traces left on the suspect’s computer. In Figure 1, the hypotheses and tests are given underneath the directed acyclic graph, which gives the possible causal relations between the hypotheses and the tests, with an arrow indicating possible causality. Note that some traces may have more than one possible cause, so that the directed acyclic graph is not a tree.

Each node in the graph can take one of three possible values: Yes, No, or Uncertain, according to whether or not the forensic analyst was able to determine the presence of absence of the specific trace. Observe that there can be actual evidence of the absence of a trace (“No” value of the node), as opposed to having no knowledge of whether the trace was ever there or not (“Uncertain”). For example, if an exterior internet source showing all uploads performed around a given time shows that the specific action of the suspect did not occur, this is evidence of absence of the trace, whereas, if the analyst cannot locate the log file on the suspect’s computer containing the list of actions performed at that time, then existence of the trace can be said to be uncertain.

We observe that, once a given test has been performed and a result obtained, it can eliminate some of the possible results from other tests, due to the complexity of the dependencies between tests that is modelled by the network structure. For example, in the Bayesian network for the Bit Torrent case given below, if a positive result is obtained on the test $E_{8},$ then the “Uncertain” value becomes impossible for the tests $E_{13}$, $E_{14}$, $E_{15}$ and $E_{17}$.

The directed acyclic graph is made into a Bayesian network by equipping it with conditional probability tables (given in the Appendix A), which indicate the probabilistic dependencies of each node on every combination of possible values of its parent nodes. The probability tables of the Bayesian network were established by interviewing 31 experienced digital forensic examiners [10].

All of our computations with the Bayesian network were made using the AgenaRisk software produced by Fenton and Neil [12].

### 4.2. Application of the Expected Information Gain Method

Let us apply the information gain method to this case, in order to determine which of the 18 possible tests should be performed first, and when to stop testing.

To apply the method, we start by equipping the Bayesian network above with *H* as the selected node, the 18 nodes $E_{1},\ldots ,E_{18}$ as the test nodes, and the rest of the network in the unfixed state. All the nodes in this network take the three possible values Yes, No, Uncertain, so we have

$$g=e_{1}=\cdots =e_{18}=3.$$

We let the indices 1,2,3 correspond to Yes, No, Uncertain in that order. We have

$$P\left(H(1)\right)=P\left(H(2)\right)=P\left(H(3)\right)=\frac{1}{3},$$

so the expression in (2) for the expected information gain on *H* given $E_{i}$ simplifies to

(6) $$I_{E_{i}}^{H}=\sum_{j,k=1}^{3}P\left(E_{i}\left(j\right)\right)P\left(H\left(k\right)|E_{i}\left(j\right)\right)\;\log_{3}\left(3\;P\left(H\left(k\right)|E_{i}\left(j\right)\right)\right).\;$$

The values of the expected information gains associated to each test, obtained by plugging the probabilities from the Bayesian network into (6), are given in the first column of Table 1 below. The only one which is significant for our purposes is the maximal one, which turns out to be $E_{8}$, with an information gain rate of 0.4083. Thus, $E_{8}$ is the test that the expected information gain method recommends should be performed first.

The next most important test would appear to be $E_{13}$, but, in fact, as noted above, the actual result of the test $E_{8}$ may change that. For example, suppose we perform the test $E_{8}$ and obtain the result Yes. Then, we redo the procedure to select the most important test on the same Bayesian network, but now equipped with the selected node *H*, the set of terminal nodes $E_{i}$ for 1≤i≤18, i≠8, and the state of the rest of the network given by setting $E_{8}=\mathrm{Yes}$. If we run the same process again, we find that the most useful next test to perform turns out to be one of the equal tests $E_{1}$ or $E_{2}$, with an information gain rate of 0.047 (these two tests will have an equal rank under any ranking method due to the fact that their conditional probability tables are identical). If we choose $E_{1}$ and assume the result is Yes, the next most useful test according to the method is then $E_{13}$, and so forth.

In Table 1, we show the results obtained when all tests are assumed to give the result Yes. Note that the initial probability for P(H) is 0.3333⋯, and that the maximal possible probability of *H*, obtained when all 18 tests have been performed and yielded the result Yes, is equal to 0.9254, so that a higher probability than 0.9254 for guilt cannot be obtained from the 18 tests alone. In order to convict the suspect, further evidence from another source may be necessary.

*Threshold of usefulness.* As noted above, the formula for the expected information gain gives a maximal possible value of 1 when the prior distribution is the equidistribution indicative of zero knowledge, as in the Bit Torrent case. Therefore, the values of the expected information gain associated to each test should be compared to the maximal possible value of 1. This gives an idea that the strongest test in the list above, $E_{8}$, is slightly less than half as strong as a test that would provide absolute certainty.

When time or resources are limited, it makes sense to stop performing tests when it is considered that the information gain is insignificant. A natural possibility is to decide to stop testing when the probability gain on the desired node drops below 1%. Thus, in the Bit Torrent case, it could be considered as not useful to perform the remaining *ten* tests which at most could raise the probability P (H = Yes) from 91.48% to 92.54%.

### 4.3. Application of the Tornado Method

We use the tornado method in the same way as the expected information gain: namely, at each stage of testing, we use the method to determine the optimal next test to perform. Assuming as above that each test performed obtains the result Yes, we obtain the results in Table 2.

We observe that the tornado method does not give a good way to define a threshold of usefulness, as the information gain method does because the increase in probability of guilt does not form a monotone decreasing sequence; for example, the increase of 0.273 at $E_{11}$ is less than the increase of 0.309 at the following step $E_{18}$. Thus, it makes little sense to stop testing when the increase drops below 1% since it may subsequently rise again. Rather, with this type of method, it is more reasonable to choose to stop at some threshold sufficiently near to the maximum result that could be obtained by getting positive results on all the tests. For instance, one could choose to stop testing once enough tests have been performed to obtain a probability of guilt within 1% of the maximum, since this means that no amount of further testing could provide more than a total of 1% increase in the probability of guilt. In the Bit Torrent example, eleven tests are necessary to reach a probability of guilt of 91.82%, which is within 1% of the maximum probability of guilt, 92.54%, which could be provided by obtaining positive results on all eighteen tests.

The results of the tornado method are strikingly different from those of the information gain method, most particularly because the test $E_{8}$ which gives the highest rate of information gain does not even appear in the list of eleven tests with the highest tornado impact. The reason for this is that the initial state of the $E_{8}$ node shows a very low probability of obtaining a positive result (9.3%), a very high probability of obtaining a negative result (51.2%) and especially a high probability of obtaining an uncertain result (39.5%) that plays no role in the tornado impact, but a significant role in the calculation of information gain. This difference between the two methods would not be as striking in the case of a Boolean network.

### 4.4. Application of the Single Missing Item Method

The single missing item method gives quite a different recommended order for testing. Like the tornado method, the increase in probability of guilt at each step is not a monotone decreasing sequence, so here again it makes more sense to choose a threshold near the maximum probability to dictate a reasonable stopping point. In the Bit Torrent example, we need to perform twelve tests, whose results are given in Table 3, to arrive at the chosen threshold of 1%.

## 5. Conclusions

In terms of digital forensic investigation strategy, searching for evidential traces with a high potential impact on the main investigation hypothesis before those of low potential impact permits unpromising investigations to be abandoned prematurely with a consequent saving of investigative resources. The question of how best to accomplish this was raised in [13] and the single missing item was introduced for this purpose in [14]. The present work recalls that method and further presents two other methods for assessing impact of tests on the final result. These methods are introduced in the most general context, as techniques applied in the fully general situation of a Bayesian network with a given distinguished node (the main investigation hypothesis) and a set of terminal nodes (tests), intended to assess the impact of each test on the main hypothesis. The expected information gain method based on the Kullback–Leibler divergence has the advantage of best reflecting the different probabilities of the possible outcomes of each test, particularly the fact that these outcomes may not be Boolean, and furthermore it provides an intrinsic threshold of usefulness below which further testing may be considered as not sufficiently useful to pursue. Such a threshold can be particularly valuable and even indispensable in cases where it is impossible to perform all the tests, for example because one test uses up all of the testable material before another can be performed, for example when choosing between testing a mixed low-template crime-scene DNA sample for Y-haplotype or for genetic profile.

In conclusion, we have demonstrated the superiority of the Kullback–Leibler divergence as a measure of impact, in the sense of information gain, over the tornado method and the single missing item method. We have shown how these three approaches can be applied to a real-world criminal case and have compared their outcomes in this actual context, noting how significantly they differ from one another. Finally, we have indicated how our results can be applied to generate more accurate, near-optimal, cost-effective forensic investigation strategies in criminal case investigations, thereby relieving somewhat the pressure on already overstretched law enforcement resources.

## Figures and Tables

**Table 1:** Tests ranked in order of expected information gain.

Test	Information Gain Method	Updated P (H = Yes)	Increase in P (H = Yes)
Prior			0.3333
$E_{8}$	0.4083	0.6829	0.3496
$E_{1}$	0.0463	0.7661	0.0832
$E_{13}$	0.0198	0.8196	0.0535
$E_{18}$	0.0060	0.8521	0.0325
$E_{4}$	0.0044	0.8803	0.0282
$E_{3}$	0.0029	0.8942	0.0139
$E_{16}$	0.0018	0.9049	0.0107
$E_{11}$	0.0014	0.9148	0.0099

**Table 2:** Tests ranked in order of tornado impact.

Test	Tornado Method Impact	Updated P (H = Yes)	Increase in P (H = Yes)
$E_{13}$	0.2913	0.6250	0.2916
$E_{3}$	0.1824	0.7226	0.0976
$E_{4}$	0.1374	0.7878	0.0652
$E_{16}$	0.0982	0.8208	0.0330
$E_{11}$	0.0735	0.8481	0.0273
$E_{18}$	0.0770	0.8790	0.0309
$E_{1}$	0.0526	0.8904	0.0114
$E_{5}$	0.0419	0.9026	0.0123
$E_{12}$	0.0246	0.9095	0.0068
$E_{15}$	0.0234	0.9148	0.0054
$E_{6}$	0.0140	0.9182	0.0033

**Table 3:** Tests ranked in order of missing item impact.

Test	Information Gain Method	Updated P (H = Yes)	Increase in P (H = Yes)
$E_{18}$	0.0633	0.5410	0.1347
$E_{13}$	0.0265	0.6759	0.0832
$E_{3}$	0.0146	0.7590	0.1283
$E_{1}$	0.0097	0.7790	0.0199
$E_{2}$	0.0097	0.7828	0.0039
$E_{4}$	0.0021	0.8361	0.0533
$E_{16}$	0.0013	0.8643	0.0245
$E_{5}$	0.0012	0.8791	0.0152
$E_{6}$	0.0012	0.8836	0.0046
$E_{8}$	0.0011	0.9118	0.0305
$E_{7}$	0.0088	0.9120	0.0003
$E_{9}$	0.0071	0.9200	0.0003

## Appendix A

In this Appendix, we give the conditional probability tables for the Bayesian network model of the Bit Torrent case. The values contained in these tables were elicited from a questionnaire survey of 31 experienced digital forensic examiners within the Technical Crime Bureau of the Hong Kong Police and the Computer Forensic Laboratory of Hong Kong Customs. The sensitivity of the posterior output of the BitTorrent Bayesian network with respect to variations in the CPT values given below has been examined previously and was found to be remarkably low [15].
